# The significance of endocannabinoid receptors and endocannabinoids in the lower urinary and genital tract

**DOI:** 10.1007/s00345-025-06045-x

**Published:** 2025-12-06

**Authors:** Stefan Ückert, Petter Hedlund, Dimitrios Tsikas, Markus A. Kuczyk

**Affiliations:** 1 Division of Surgery, Department of Urology & Urological Oncology, Hannover Medical School, 30623 Hannover, Germany; 2 Faculty of Medicine, Institute of Laboratory Medicine, Department of Clinical Pharmacology, Linköping University, Linköping, Sweden; 3 Hannover Medical School, Institute of Toxicology, Core Unit Proteomics, Hannover, Germany

**Keywords:** Endocannabinoid system (ECS), Urinary tract, Human male genital tract

## Abstract

**Purpose:**

The cannabinoid receptor (CB) signal transduction is a complex system that is relatively widespread in mammalian cells and tissues. By regulating the local levels and actions of other chemical signals, it can activate multiple cascades in different organs. Cannabinoid receptors and endocannabinoids are also present in the human lower urinary tract (LUT) and genital tract, including the urinary bladder, prostate, urethra, penile erectile tissue and seminal vesicles. The present review aims to give an overview on what has emerged from basic research and clinical approaches on the role of the endocannabinoid system (ECS) in the human lower urinary tract and reproductive tissues.

**Methods:**

This review article highlights some key data from research into the cannabinoid system and its potential role in the control of micturition, reproductive functions and also dysfunctions of the lower urinary tract.

**Results:**

While basic research findings on the ECS in reproductive tissues (penile erectile tissue, seminal vesicles) have not yet been supplemented by data from trials addressing clinical conditions such as erectile dysfunction (ED) and ejaculatory disorders (for example, premature ejaculation), targeting the ECS has been suggested as a pharmacological approach to treat disorders of bladder filling and storage functions.

**Conclusion:**

Despite some preclinical data may favor a role of receptors, ligands and regulatory proteins of the ECS in the LUT, additional clinical evidence is required in order to support a significance of a modulation of the pathways stimulated by cannabinoid molecules in the treatment of dysfunctions of the lower urinary and genital tract.

## Introduction

**Fig. 1:**
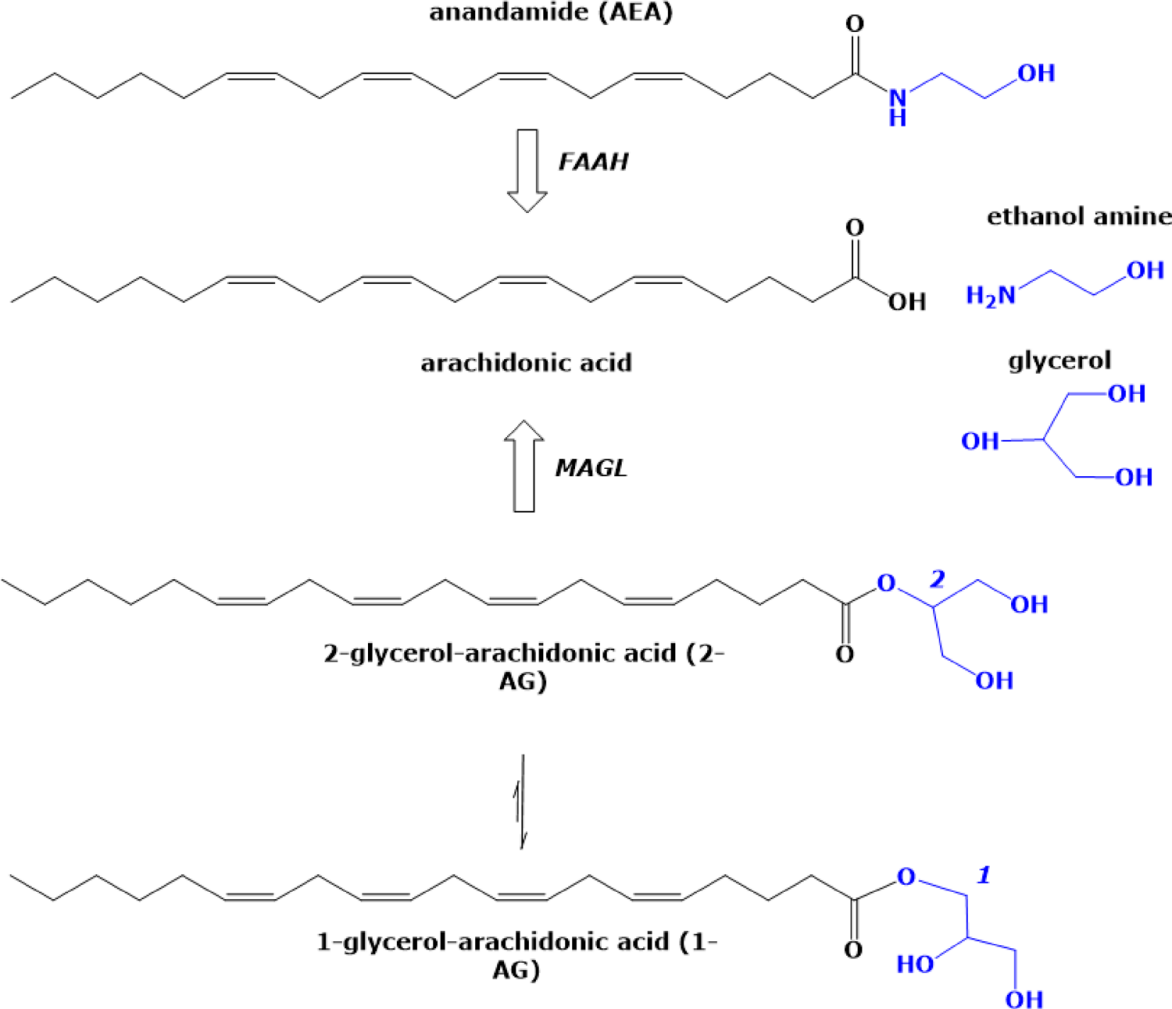
Chemical structures of the endocannabinoids anandamide (AEA) and 2-glycerol-arachidonic acid (2-AG) and their enzymatic hydrolysis by fatty acid amide hydrolase (FAAH) and monoacylglycerol lipase (MAGL) to arachidonic acid and glycerol, respectively. 2-glycerol-arachidonic acid is non-enzymatically converted to 1-glycerol-arachidonic acid (1-AG)

In the previous decades since its discovery, the pleiotropic endogenous cannabinoid system (ECS) has been implicated to trigger many physiological processes, and the signaling pathways mediating many of these responses, particularly in the central nervous system, have come into light. It has become obvious that the cannabinoid receptor signal transduction is a complex system that is relatively widespread in mammalian tissues and cells and can activate, by regulating the local levels and actions of other chemical signals, multiple cascades in different organs including those of the lower urinary and genital tract of humans. This system comprises of at least two G-protein-coupled receptors, known as the cannabinoid receptors CB1 and CB2, and endogenous agonists of these receptors, known as endocannabinoids, of which anandamide (*N*-arachidonoyl ethanolamide = AEA), 2-arachidonoyl glycerol (2-AG), palmitoyl ethanolamide (PEA) and oleoyl ethanolamide (OEA) are the most important. AEA is known to attach with higher affinity to the CB1 receptor [HS, IM]. OEA and PEA mainly act as entourage compounds by inhibiting the hydrolysis of AEA by the enzyme fatty acid amide hydrolase FAAH [HS]. The non-endogenous cannabinoid delta^9^-tetrahydrocannabinol (Δ^9−^THC) is the major psychoactive constituent of the plant *Cannabis sativa* and is characterized by a partial agonist activity at the cannabinoid receptor CB1, located mainly in the central nervous system. FAAH hydrolyzes anandamide into arachidonic acid and ethanolamine while monoacylglycerol lipase (MAGL) hydrolyzes 2-AG to arachidonic acid and glycerol, thus facilitating regulation of the levels of endocannabinoids [1]. In the human lower urinary and genital tract, immunoreactivity for CB1 and CB2 has been localized in the penile erectile tissue (corpus cavernosum penis) and to nerve fibers of the urethra, and the expression of CB2 receptors, suggested to have a role in sensory (neuro)signaling, has been reported in the mucosa of the urinary bladder [2–4]. The number of members of this endocannabinoid signaling system still seems to increase, as new non-CB1 and non-CB2 receptors, endocannabinoid-related molecules with little activity at the CB1 and CB2 receptors, as well as new enzymes for the biosynthesis and degradation of endocannabinoids have yet been identified [5]. However, at the time of writing this manuscript, it still remains unclear whether compounds that can selectively interfere with the levels of endocannabinoids or their actions at CB receptors may potentially represent promising templates for new drugs to address dysfunctions of the lower urinary and also the genital tract. More conclusive evidence of the significance of molecular pathways stimulated by anandamide and other cannabinoid molecules is required. The present review aims to briefly summarize what has emerged from basic research and clinical approaches on the role of the ECS in the human LUT and in genital tissues Fig.1.

### Lower urinary tract (urinary bladder, urethra)

Numerous studies using tissue specimens from rodents, monkeys and also man have identified the presence of CB receptors, AEA, 2-AG, endocannabinoid-like compounds (e.g., fatty acid ethanolamides), the AEA-producing enzyme N-acyl-phosphatidyl ethanolamine, as well as FAAH and MAGL in the LUT and in neuronal pathways involved in the control of micturition [6–8]. A role for the control by CB1 of neurons mediating autonomic efferent innervation in bladder tissues has been suggested from previous investigations, but seems to vary significantly between species [9, 10]. Mechanistically, it has been proposed that CB1 receptors modulate transmitter release from motor neurons in the bladder through presynaptic regulation. However, without any relevant data from experiments directly measuring the release of acetylcholine in the tissue before and after CB1 signal modulation, this remains speculative. Furthermore, the modest inhibitory effects of the selective CB1 agonist ACEA on electrically induced contractions in bladder tissue excised from humans do not convincingly support a significance of CB1-mediated modulation of bladder efferent nerve fibers [11]. In mouse and rat bladder tissue, inhibitory effects of CB agonists on nerve-induced contractions have been shown to be attenuated by antagonism of CB2 [9]. In contrast, in rat bladder tissue, the CB2-selective agent cannabinor had only minimal effects on contractions induced via transmural activation of nerves and also had no impact on the release of ³H-choline from nerve endings [12]. Overall, there is insufficient evidence to support the presynaptic modulation via CB1- or CB2-mediated mechanisms of the release of transmitters from cholinergic motor nerves in the bladder. More supportive experimental evidence advocates a relevance for the ECS in modifying afferent sensory functions in the LUT. CB1 or CB2 receptors are located in the urothelium and in sensory nerve terminals of the bladder and urethra, as well as in dorsal root ganglia supplying the LUT. In humans, CB1 receptors have been located to the lumbar superficial dorsal that contains afferent neurons supplying the LUT [13, 14]. Co-localization of CB1 or CB2 receptors with sensory markers, e.g., the calcitonin gene-related peptide (CGRP), subtypes of the transient receptor potential cationic channel (TRPA1, TRPV1) or adenosine triphosphate (P2 × 3) receptors, have been described in these structures [6, 8, 14]. CB agonists have been shown to counteract the release of CGRP in isolated mouse bladder tissue and to reduce firing of afferents axons in ex vivo whole bladder preparations from rat and guinea-pig [15, 16]. In anesthetized rats, during the phase of filling of the bladder (as assessed by cystometry), recordings of afferent nerve activity were increased by either CB1 or CB2 antagonism or reduced by a FAAH inhibitor [17]. In fact, compounds targeting the ECS have been reported to exhibit effects in rodent models of bladder disease. In cases of bladder overactivity resulting from outflow obstruction, the CB2 agonist cannabinor or the inhibition of FAAH have been shown to reduce bladder overactivity and increase bladder capacity and intervals of micturition [12, 18].

Assuming alterations of the ECS and its functions in humans with painful bladder syndrome (associated with bladder inflammation), Mukerji et al.. (2010) reported an altered expression of the density of the CB receptor in correlation to symptoms. However, these findings were based on a visual (histological) grading method and were not supported by the biochemical measurement of protein levels [19]. In addition, inconsistent data on the levels of expression of the CB receptor and also anandamide, 2-AG and endocannabinoid-like compounds (whether unaltered, decreased, or increased) in bladder tissue originating from models of experimental cystitis highlights the need for further investigations into the potential of the ECS as a therapeutic target in bladder inflammation to achieve reduction of bladder reflex hyperactivity and visceral pain and an increase in micturition intervals and bladder capacity [20]. In a study involving 255 patients with LUTS due to multiple sclerosis (CAMS-LUTS Study), treatment with THC resulted in a significant reduction in the episodes of urge incontinence when compared to the placebo group. However, a sub-analysis of a small number of patients revealed no urodynamic changes [21]. In related study protocols, nabiximols, a combination of cannabidiol (CBD) and THC licensed for spasticity associated with MS, showed a (non-significant) trend towards reducing urgency episodes and was also reported to reduce nocturia, ameliorate bladder symptoms (bladder symptom severity score) and exert beneficial effect on post-void residual urine volume [22–24]. Overall, although some preclinical data seem to favor a role for the ECS in normal micturition and in models for LUT inflammation, the information from clinical trials is not always consistent and there still is insufficient understanding of the molecular basis of the involvement of this pathway in LUT function and dysfunction. Also, from the translational perspective, it should be taken into consideration that merits on the clinical effects by cannabinoids on LUTS in humans are almost solely derived from patients with MS. Moreover, one should keep in mind that the clinical outcomes of the studies might be, to a certain degree, affected by the limitations brought about by the urodynamic testings performed (for example, the effect of urine filling rate on bladder sensation at a certain volume, with bladder filling rates of > >10 ml/min possibly evoking a higher local release of ATP). This may also account to the discrepant findings from studies using the combination of cannabidiol and THC. In fact, additional large, high-quality clinical studies are necessary to consistently investigate across all trials efficacy endpoints in LUTS [25].

### Prostate

It has been known for several years that CB1, FAAH and other components of the ECS are expressed in the human prostate gland. In prostate cancer (PC), one of the most common malignant diseases in men, a dysregulation of the ECS as an underlying cause has been implicated. Pronounced alterations in the expression of distinct constituents of the ECS have been found to correlate to the malignant grade of the solid tumor, tumor progression and, thus, to the clinical outcome of the cancer [26]. For example, the expression of CB1 and CB2 is increased in PC, and a high expression of CB1 has been associated with poor prognosis for the patients. Indeed, in in vitro settings, some cannabinoids have shown a high anti-cancer efficacy in PC, but the specific molecular mechanisms mediating the effects observed have not yet been elucidated comprehensively with regard to the drugs used and the tumor subtypes that were examined [27, 28]. In a cohort study conducted in 1812 men with PC to investigate by means of LC-MS/MS the serum concentrations of a total of 49 metabolites, including arachidonic acid, AEA and 2-AG, inoleoyl ethanolamide, *N*-oleoylserine and *N*-stearoylserine, neither AEA nor 2-AG were found to be associated with prostate cancer-specific survival. The investigators reported elevated concentrations of linoleoyl ethanolamide, *N*-oleoylserine and *N*-stearoylserine, however, it has been assumed that these compounds cannot be assigned as endocannabinoids [29].

It has been reported that the CB2 receptor can form heteromeric structures with the GPC chemokine receptor CXCR4 in PC cells. CXCR4 can enhance the ability of cells to proliferate and migrate into other tissues. The heteromerization with CB2 might finally enable cannabinoid compounds to exert reduction of the invasive properties of cancer cells by inhibiting the effects of endogenous agonists of CXCR4, and may represent a novel pharmacological tool to affect tumour cell migration and invasion (such as the growth of metastasis) [30]. While some authors consider endogenous and exogenous cannabinoids as upcoming attractive agents bearing the pharmacological potential to target PC, others have emphasized that, up until today, there is insufficient data to support the claim for cannabinoids as an anti-cancer treatment option. This is supported by the fact that at least no data from clinical trials are at present available to add reasonable and convincing evidence to the results that emerged from in vitro or animal studies suggesting that cannabinoids may provide therapeutic benefits to anti-cancer treatment strategies [31]. In general, the translation of findings from in vitro studies and the measurement by biochemical methods (for example, LC-MS/MS) of marker compounds in body fluids into the most complex entity of a dynamically proceeding cancerous disease in humans is rather challenging.

With regard to non-malignant (benign) deteriorations of the prostate, as seen in lower urinary tract symptomatology (secondary to benign prostatic enlargement/hyperplasia), increased levels of palmitoyl ethanolamide (PEA) and arachidonoyl ethanolamide (AEA) (as determined by LC-MS/MS, followed by multivariate analysis) were registered in the blood serum of a small cohort of 66 older men (aged 65 to 80 years) suffering from nocturia (in comparison to age-matched healthy subjects, control) [32]. The observation, as assessed by Western blotting, that nitrinergic/cholinergic nerves (that stained positive for NOS and the vesicular acetylcholine transporter) containing immunoreactivity for CB1 and CB2 were scarce or even absent in tissue samples from verified histopathological nodular prostate hyperplasia when compared to non-benign tissue has yet not been linked to any correlate that may bear potential clinical significance [33]. In an animal study conducted in male rats with metabolic syndrome (MetS) induced by feeding the animals for 8 weeks a concentrated fructose solution and high salt diet (delivered as food pellets), signs of BPH (prostate weight as well as histopathological indicators, such as interleukin 6 and the proliferation marker cyclin D1) significantly decreased when administering to the animals the CB1 antagonists AM 6545 or AM 4113 (10 mg/kg/day). When compared to the untreated group (MetS), rats treated with the CB1 antagonists had reduced concentrations of AEA and 2-AG in the prostates. It was concluded, that CB1 antagonism may serve to protect against BPH, induced by metabolic disorders (such as MetS), probably via anti-proliferative and anti-inflammatory effects [34]. In a clinical setting, 229 adult male patients with verified chronic prostatitis and/or chronic pelvic pain syndrome (CP/CPPS) were randomized to various doses of the FAAH inhibitor ASP 3652 or to placebo for at least 12 weeks. ASP 3652 did not show significant efficacy on the pain symptoms reported by the patients, however, despite the fact that some findings were not straightforward, the authors concluded that their results may hint to an attenuation of LUTS (potentially triggered by the immanent inflammatory process) through the inhibition of FAAH activity [35].

### Seminal vesicles (VS)

In the male reproductive tract, the seminal vesicles (SV) are involved in the process leading up to seminal emission and ejaculation. There is extensive evidence that the normal function of SV smooth musculature, in particular the contractile activity, contributes to the facilitation of this process [36]. Hence, It has been suggested that disturbances on the level of the neuromuscular control may result in ejaculatory dysfunctions, such as anejaculation or premature ejaculation (PE) [37, 38]. Beside erectile dysfunction (ED), PE is the most common sexual disorder among adult males. The pharmacotherapy of PE has focused primarily on topical anaesthetics (prilocaine/lidocaine) and selective serotonin (5-HT) re-uptake inhibitors (SSRI), such as clomipramine, fluoxetine, imipramine, paroxetine, sertraline or, but mainly, dapoxetine, administered orally [39–41]. However, the community of pharmacologists still aims to identify new options for the pharmacological treatment of PE. This takes into account the administration of drugs affecting the sympathetic, motor pudendal or suprasacral control of the ejaculation reflex, the activity of serotonin receptors, as well as the contraction and relaxation of the smooth musculature of both the SV and ductus deferens [42]. Recently, a role for the ECS in ejaculatory function has been considered, as AEA, an endogenous cannabinoid receptor ligand, was seen to target the threshold for ejaculation in male sluggish rats [43]. In the human SV, by means of molecular biology (RT-PCR), PCR products corresponding to the cannabinoid receptors CB1 and CB2, GPR55 (an orphan G protein-coupled receptor that recognizes cannabinoid ligands), as well as to the FAAH isoenzymes 1 and 2 have been detected. Immunohistochemistry revealed dense expression of CB1, CB2 and GPR55 located to the pseudo-stratified columnar glandular epithelium and also in varicose nerves interspersing the epithelium. These nerve fibers (assumed to be sensory/parasympathetic nerves) were also characterized by the expression of the neuropeptides vasoactive intestinal polypeptide (VIP) and calcitonin gene-related peptide (CGRP). Cytosolic staining for FAAH1 and FAAH2 was seen in cuboidal cells of all layers of the epithelium while no immunoreactivity was detected in the smooth musculature or in slender nerve fibers [44]. The predominant expression of CB1, CB2, GPR55 and FAAH1 and 2 in the epithelial layer shows evidence that these proteins are more likely to be involved in the control of secretory function than in neuromuscular signaling. The hypothesis of an involvement of the ECS in epithelial homeostasis is strongly supported by the observations that AEA and 2-AG are markedly reduced in the seminal plasma of infertile men [45]. However, since no functional experiments have been conducted till now, the potential role of the ECS in mechano-afferent/efferent signaling remains to be elucidated. Considering the locations of immunoreactivity exemplified above, the ECS may rather be involved in epithelial homeostasis and secretory function of the SV. In fact, the findings provide a basis to further investigate whether the modulation of the ECS has a relevant effect on nerve-mediated functions, including autonomic mechano-efferent signaling, in the ejaculatory duct.

Up until today, data on a potential significance of the ECS in the vas deferens have been limited to animal studies. In tissue bath experiments using epididymal vas deferens isolated from rats, the contraction response of the tissue elicited by means of electrical field stimulation (single pulses, 150 V, 0.5 ms, delivered every 30 sec) was inhibited by increasing concentrations of the cannabinoid agonists WIN 55.212-2.212 or CP 55.940 (presumably acting on prejunctional cannabinoid CB1 receptors). The response curves were shifted towards an enhancement of tissue contraction by the selective CB1 antagonists SR 141,716 A or LY 320,135 [46]. Direct relaxing effects of anandamide (up to 10 µM) on the contraction responses of rat vas deferens mediated by calcium chloride have also been reported. The CB2 antagonist AM 630 significantly reversed the (Ca^2+^ antagonistic) relaxation response [47].

In light of these findings, it seems that, in the vas deferens, agonist/antagonist interactions at both types of CB receptors trigger different signaling mechanisms. Further studies are needed to elucidate the signaling mechanism of action of anandamide.

### Penile erectile tissue (Corpus cavernosum penis)

Penile erection in response to sexual stimulation requires cavernous arterial vasodilatation, the relaxation of trabecular smooth muscle, brought about by the interaction of various transmitters and effector compounds acting in the central nervous system (brain, spinal cord), or produced locally either by neuronal or endothelial structures, and a reduction of venous outflow, the so-called veno-occlusive mechanism. Numerous basic studies on the mechanism of penile erection have broadened the knowledge on this process. Detailed information on erectile physiology and pathophysiology has been gained and extensive coverage was made on the principles of erectile neurotransmission, especially on the nitric oxide (NO)/cyclic GMP pathway. Research on male erectile dysfunction (MED) has primarily focused on central and peripheral mechanisms of corpus cavernosum smooth muscle relaxation, which is the crucial step in achieving an erection [48–50]. Based on the knowledge on intracellular signal propagation in cavernous smooth muscle, the treatment of MED has evolved from the intracavernosal or intraurethral application of vasoactive drugs to the recent use of oral therapies (phosphodiesterase type 5 inhibitors) acting via maximizing the NO/cyclic GMP signaling [51, 52]. Although it is without doubt that the release of the gaseous transmitter molecule NO from nitrinergic nerve fibers is essential in the control of penile erection, it still remains to be clarified whether other non-adrenergic/non-cholinergic (NANC) factors are also involved in the maintenance of corpus cavernous smooth muscle tone. Such compounds may include endogenous bioactive peptides, such as the atrial natriuretic peptide (ANP), C-type natriuretic peptide (CNP), vasoactive intestinal polypeptide (VIP) and calcitonin gene-related peptide (CGRP) [53, 54], and also mediators of the ECS. Using isolated rat penile erectile tissue, Ghasemi et al. (2006) demonstrated by means of Western blotting the existence of CB1 receptors. In tissue bath experiments, the endogenous cannabinoid agonist anandamide (3 µM) significantly enhanced the NANC relaxation dependent on NO (sensitive to pre-incubation with the NOS inhibitor L-NAME) mediated by electrical field stimulation (EFS, at frequencies of 8 Hz to 32 Hz) of tissue strips challenged by the adrenergic agonist phenylephrine (PE). The response was significantly attenuated by the selective cannabinoid CB1 receptor antagonist AM 251 and the vanilloid receptor antagonist capsazepine (3 µM each) but not the selective CB2 antagonist AM 630. No effects of anandamide were registered on concentration-dependent relaxation responses exerted by the NO donor drug sodium nitroprusside (SNP, 10 nM to 1 mM) [55].The authors concluded that the enhancing effect of anandamide on the relaxation mediated by NANC factors of rat corpus cavernosum subjected to EFS involves activation of both the CB1 and vanilloid receptors. Gratzke et al. (2010), using immunohistochemical methods, were the first to demonstrate the presence of CB1 and CB2 in the human corpus cavernosum. Immunoreactivity (IR) was found mainly in nerve fibers that were also characterized by the expression of nitric oxide synthase (NOS) or the transient receptor potential cationic channel V1 (TRPV1), thus indicating potential interactions between the ECS and the signal transduction mediated by NO. In the nerves captured, IR for CB1 or CB2 was not colocalized with signals specific for CGRP or tyrosine hydroxylase [2]. Investigations were also conducted on the effects of the cannabinoid receptor agonists anandamide, arachidonyl-2-chloroethylamide (ACEA) or JHW 015 and the antagonists AM 251 or AM 630 on NANC relaxations evoked by EFS of isolated rabbit corpus cavernosum. In these experiments, nitrinergic relaxation responses of the tissue (precontracted by an adrenergic agonist) were blocked by the presence of the NOS inhibitor L-arginine methylester (L-NAME). Anandamide, ACEA and JHW 015 enhanced the non-nitrinergic NANC relaxations whereas these relaxation responses were significantly inhibited by AM 251 or AM 630. Anandamide did not alter the non-nitrinergic NANC relaxations brought about by EFS in the presence of AM 251 or AM 630. These results may suggest that, in human penile erectile tissue, non-nitrergic NANC relaxations are mediated in part by cannabinoid-like factors that are released following excitation from non-nitrinergic nerves and assumed to act at both the cannabinoid receptors CB1 and CB2 [56].

## Concluding remarks

When compared to other pharmacological principles applied in clinical routine to patients with urinary tract disorders or sexual dysfunctions, only very little knowledge has been gained on the potential efficacy of pharmacological interference with the ECS to treat disorders of the lower urinary tract and sexual dysfunctions in both sexes. While, for example, there are substantial data on the use of phosphodiesterase type 5 (PDE5) inhibitors to treat male erectile dysfunction, on alpha 1-adrenoceptor antagonists or inhibitors of the enzyme 5-alpha reductase to ameliorate LUTS (secondary to benign prostatic enlargement), on selective serotonin (5-HT) re-uptake inhibitors to target premature ejaculation (PE) in men, and on the use of anti-muscarinic agents in bladder overactivity, at the time of writing this manuscript, no natural or synthetic agonist of the CB1 or CB2 receptor (AEA, 2-AG, THC, cannabinor) or an inhibitor of the enzyme FAAH (such as oleoyl ethylamide or ASP 3652) has been incorporated into routine application in the field of urology. This is due to the fact that, with regard to normal and diseased tissues of the urinary and genital tract, there are still many conflicting data or inconsistent findings on the levels of endocannabinoid-like compounds and/or the expression of CB receptors (whether unaltered, decreased or increased). However, although urodynamic studies conducted in rodents consistently report that CB agonists affect parameters related to sensory functions during bladder filling and micturition, findings from animal models do not necessarily predict beneficial effects of endogenous molecules acting at CB receptors in bladder pathophysiology (storage and voiding disorders). Extended data from large scale clinical trials in humans are still not available. This highlights the need for further investigations into the potential of the ECS as a therapeutic target in non-malignant and malignant urological disease patterns. Overall, also some preclinical data favor a role of the ECS in the LUT, the information disclosed is not always consistent and well founded, and should be further explored in order to understand better the involvement of this particular pathway in LUT function and dysfunction. Future insights from preclinical and clinical pharmacology may add to assess and validate whether the receptors, ligands and regulatory proteins of the ECS represent an intriguing pharmacological approach in selected populations of patients.

## Data Availability

No datasets were generated or analysed during the current study.
